# Sports activity participation and subjective health status of patients after total hip arthroplasty via the anterolateral-supine approach: a case series study

**DOI:** 10.1186/s12891-022-05886-6

**Published:** 2022-10-29

**Authors:** Yoshiki Takahashi, Naonobu Takahira, Katsufumi Uchiyama, Kensuke Fukushima, Mitsutoshi Moriya, Manaka Shibuya

**Affiliations:** 1 Sensory and Motor Control, Graduate School of Medical Sciences, Kitasato University, 1-15-1 Kitasato, Minami-ku, 252-0373 Sagamihara-city, Kanagawa Japan; 2 Department of Orthopaedic Surgery of Clinical Medicine, Rehabilitation Sciences and Functional Restoration, Science of Sensory and Motor Control, Graduate School of Medical Sciences, Kitasato University, 1-15-1 Kitasato, Minami-ku, 252-0373 Sagamihara-city, Kanagawa Japan; 3 Department of Rehabilitation, School of Allied Health Sciences, Kitasato University, 1-15-1 Kitasato, Minami-ku, 252-0373 Sagamihara-city, Kanagawa Japan; 4 Department of Patient Safety and Healthcare Administration, School of Medicine, Kitasato University, 1-15-1 Kitasato, Minami-ku, 252-0374 Sagamihara-city, Kanagawa Japan; 5 Department of Orthopaedic Surgery, School of Medicine, Kitasato University, 1-15-1 Kitasato, Minami-ku, 252-0374 Sagamihara-city, Kanagawa Japan; 6 Department of Rehabilitation, Kitasato University Hospital, 1-15-1 Kitasato, Minami-ku, Kanagawa 252-0375 Sagamihara-city, Japan

**Keywords:** Total hip arthroplasty, Anterolateral-supine approach, Sports activity, Subjective health status

## Abstract

**Background:**

No reports have been published about participation in sports activity and subjective health status after total hip arthroplasty via the anterolateral approach in the supine position (ALS-THA) in Japanese patients. This study assessed sports activity participation and subjective health status, as well as factors potential associated with these variables, in patients who underwent ALS-THA.

**Methods:**

Of 698 patients who underwent total hip arthroplasty at our institution between 2013 and 2018, questionnaires were sent to 355 patients under 80 years old who had undergone ALS-THA and 242 responded. Patients were asked about their subjective health status, participation in sports activity, the EuroQol 5-dimensions 5-level (EQ-5D-5L), the University of California Los Angeles (UCLA) activity scale score and the Forgotten Joint Score (FJS). Patient characteristics and hospitalization information were also collected. Patients’ subjective health status was categorized as “healthy” or “unhealthy”. Univariate and multivariate logistic regression analyses were performed to determine factors associated with participation in sports activity after ALS-THA and a “healthy” status.

**Results:**

The pre- and postoperative sports activity participation rates were 54.0% and 57.8%, respectively. Most patients (76.8%, *n* = 182) were considered “healthy”. Age (*P* = .019) and UCLA activity score (*P* < .001) were significantly associated with sports activity after ALS-THA. FJS (*P* = .002) and EQ-5D-5L (*P* = .004) were significantly associated with a “healthy” status.

**Conclusion:**

Patients participating in sports activity after ALS-THA are older and have higher UCLA activity scores and patients considered “healthy” have higher FJS and EQ-5D-5L scores.

**Supplementary Information:**

The online version contains supplementary material available at 10.1186/s12891-022-05886-6.

## Background

The “red zone” is the difference between life expectancy and healthy life expectancy (HLE) [1]. Reducing this period—that is, extending HLE—is important worldwide. Limiting sports activity reduces not only physical activity, but also quality of life and HLE [2]. Meanwhile, participation in sports activity is associated with lower mortality rates in middle and old age [3]. Furthermore, mortality related to lack of exercise is more severe than that related to obesity [4]. Accordingly, orthopaedic diseases are reported to limit physical activity [5] and hence impair HLE.

Total hip arthroplasty (THA) is a well-accepted treatment for hip osteoarthritis (OA) that can improve physical function and quality of life [6]. Bayliss et al. reported that the 20-year survival rate after THA for patients over 60 years old is 89.7% [7]. In addition, the long-term mortality rate of elderly patients who have undergone THA appears to be lower than that of elderly people from the general population [8]. Patients expect to participate in sports activity postoperatively [9]. A previous study investigated sports activity before and after THA via the posterior and lateral approach [10]. THA via the anterolateral approach in the supine position (ALS-THA) is performed using a minimally invasive approach based on the anterior Watson-Jones approach and, when compared to THA via the conventional posterior or lateral approach, ALS-THA yields better outcomes in terms of physical function and earlier discharge from hospital [11].

Despite the reported benefits of ALS-THA, no reports have been published about sports activity participation and subjective health status in Japanese patients who have undergone ALS-THA. Scott et al. investigated health status during the waiting period before surgery using the EuroQol 5-dimensions 5-level (EQ-5D-5L) score, and reported that a health status “worse than death” was associated with worse outcomes after surgery [12]. In our previous study on patients who underwent curved periacetabular osteotomy, we reported that 72.1% participated in sports activity postoperatively, and 74.1% and 38.8% of patients were “very satisfied” or “somewhat satisfied” with their current life and sports activity, respectively [13]. Therefore, this study examined sports activity participation, satisfaction with daily life and sports activity, factors potentially associated with postoperative participation in sports activity and subjective health status in patients who underwent ALS-THA, as well as factors potentially associated with subjective health status.

## Methods

### Study design and patients

**Fig. 1:**
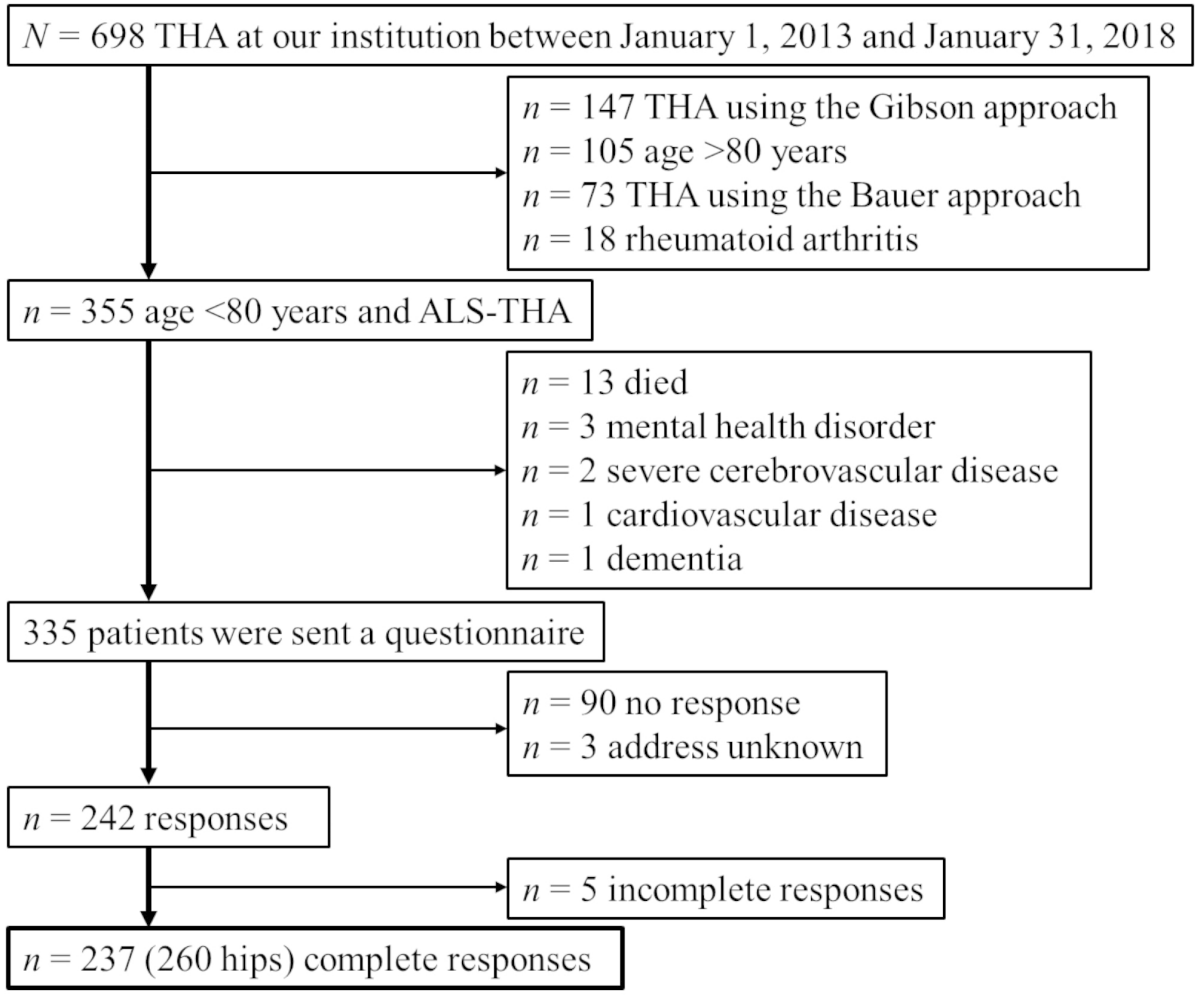
Patient enrollment flowchart. ALS-THA, total hip arthroplasty via the anterolateral-supine approach in the supine position; THA, total hip arthroplasty

This retrospective case series study was performed with approval from our institutional ethics committee. Among 698 patients who underwent primary THA at our institution between January 1, 2013, and January 31, 2018, 355 with OA (297 patients) or idiopathic femoral head necrosis (58 patients) who were younger than 80 years and underwent ALS-THA were recruited. Four surgeons made the diagnosis using radiographs and performed operations for the 355 patients. Patients who died or had mobility-limiting conditions, including severe cerebrovascular disease, cardiovascular disease, mental health disorders and dementia, were excluded. The remaining 335 patients were sent a questionnaire on their sports activity, to which 242 (72.9%) responded. However, 5 patients provided incomplete responses to the questionnaire, so 237 patients were finally included in this study (Fig. 1). All patients provided informed consent to participate in this study as per the instructions enclosed with the questionnaire.

### Questionnaire (Additional file 1)

In this study, participation in sports activity was defined as voluntary exercise. Sports activity was categorized as low, intermediate or high impact, as previously described [14]. The questionnaire comprised four sections. In the first section, patients were asked about their subjective health status [15]. The second section surveyed participation in sports activity and satisfaction with daily activities and sports activity before ALS-THA. Patients were specifically asked about whether they participated in sports activity during the 3 years before ALS-THA, the types and frequencies thereof, and their satisfaction with daily life and sports activity. The third section surveyed participation in sports activity after ALS-THA. Patients were also asked about whether they participated in sports activity as well as the types and frequencies thereof. If a patient did not participate in sports activity, they were asked why and whether they hoped to do so. The fourth section surveyed current satisfaction with daily life and sports activity, the University of California Los Angeles (UCLA) activity score [16, 17], the Forgotten Joint Score (FJS) [18], the Japanese version of the EQ-5D-5L [19, 20] and the EQ Visual Analogue Scale (EQ-VAS) [19, 20]. The EQ-VAS ranges from 0 to 100, corresponding to “worst imaginable health state” and “best imaginable health state”, respectively.

### Patient information

The following demographic and clinical characteristics were collected from medical records: age at surgery, sex, body mass index, hospitalization duration, range of motion, lower-leg muscle strength, 10-m gait speed and Japanese Orthopaedic Association Hip-Disease Evaluation Questionnaire (JHEQ) [21]. Range of motion, lower-leg muscle strength and 10-m gait speed (comfortable and maximum) were assessed by physical therapists 1-2 days before surgery and before discharge. The JHEQ consists of three subscales: pain, movement and mental; higher scores indicate higher quality of life.

### Operative technique and postoperative rehabilitation protocol

The surgical technique was a version of the ALS-THA technique reported by Pfluger et al. modified by an orthopedic surgeon (one of the co-authors: NT). Specifically, the modification was to make oblique skin incisions in both skin and fascia to create a figure-4 shape on both lower limbs. Therefore, even if the lower limb is extended after the implant is installed, no extra incision is needed. The retractor to the wound is a special retractor holder that includes weights that maintain muscle elasticity. As a Y-zone process, the distal fascia is clearly divided between the vastus lateralis and gluteus medius. Then, after the THA is placed, because the bilateral lower limbs have already been disinfected and protected with elastic bandages, it is easy to confirm the accurate correction of the leg length discrepancy of the bilateral lower limbs, which is more clinically appropriate. In addition, all patients had a drain placed in the affected THA area. Postoperative rehabilitation was performed in a wheelchair on the first postoperative day, while gait training started the day after. Most patients were discharged home 10 to 14 days postoperatively. However, 38 patients who wanted to continue rehabilitation or had difficulties that prevented discharge home were transferred to a rehabilitation hospital.

### Statistical analysis

The analysis in this study was based on 237 patients who completed the questionnaire (Fig. 1). Continuous variables are presented as mean ± standard deviation (SD). McNemar’s test was performed to compare pre- and postoperative sports activity participation rates as well as satisfaction with daily life and sports activity. Patients who participated in sports activity postoperatively and who did not were categorized into the participation and nonparticipation groups, respectively. In addition, patients were categorized into the “healthy” and “unhealthy” groups according to the questionnaire results [15]. Specifically, patients who indicated that their subjective health was affecting their daily life or that they were not “healthy” were classified as “unhealthy”, whereas the others were classified as “healthy”. To determine the factors associated with postoperative participation in sports activity and a “healthy” status, univariate and multiple logistic regression analyses were performed. The following variables were included in the multiple logistic regression analysis to determine the factors associated with postoperative participation in sports activity: age, sex, body mass index, comfortable 10-m gait speed at discharge, FJS, EQ-5D-5L, EQ-VAS and UCLA activity score. Meanwhile, the following variables were included in the multiple logistic regression analysis to determine the factors associated with a postoperative “healthy” status: preoperative hip abductor muscle strength, preoperative comfortable 10-m gait speed, FJS, EQ-5D-5L and UCLA activity score. All statistical analyses were performed using IBM SPSS Statistics version 26 (IBM Corp., Armonk, NY, USA). *P*-values less than 0.05 were considered statistically significant.

## Results

The questionnaire was filled out 34.3 ± 11.8 months from the date of surgery. The mean age of patients who responded to the questionnaire was 64.0 ± 9.7 years (range 23–79). The pre- and postoperative sports activity participation rates were 54.0% and 57.8%, respectively. Among the patients who participated in sports activity preoperatively, 82.8% of them returned to sports activity postoperatively.

**Fig. 2:**
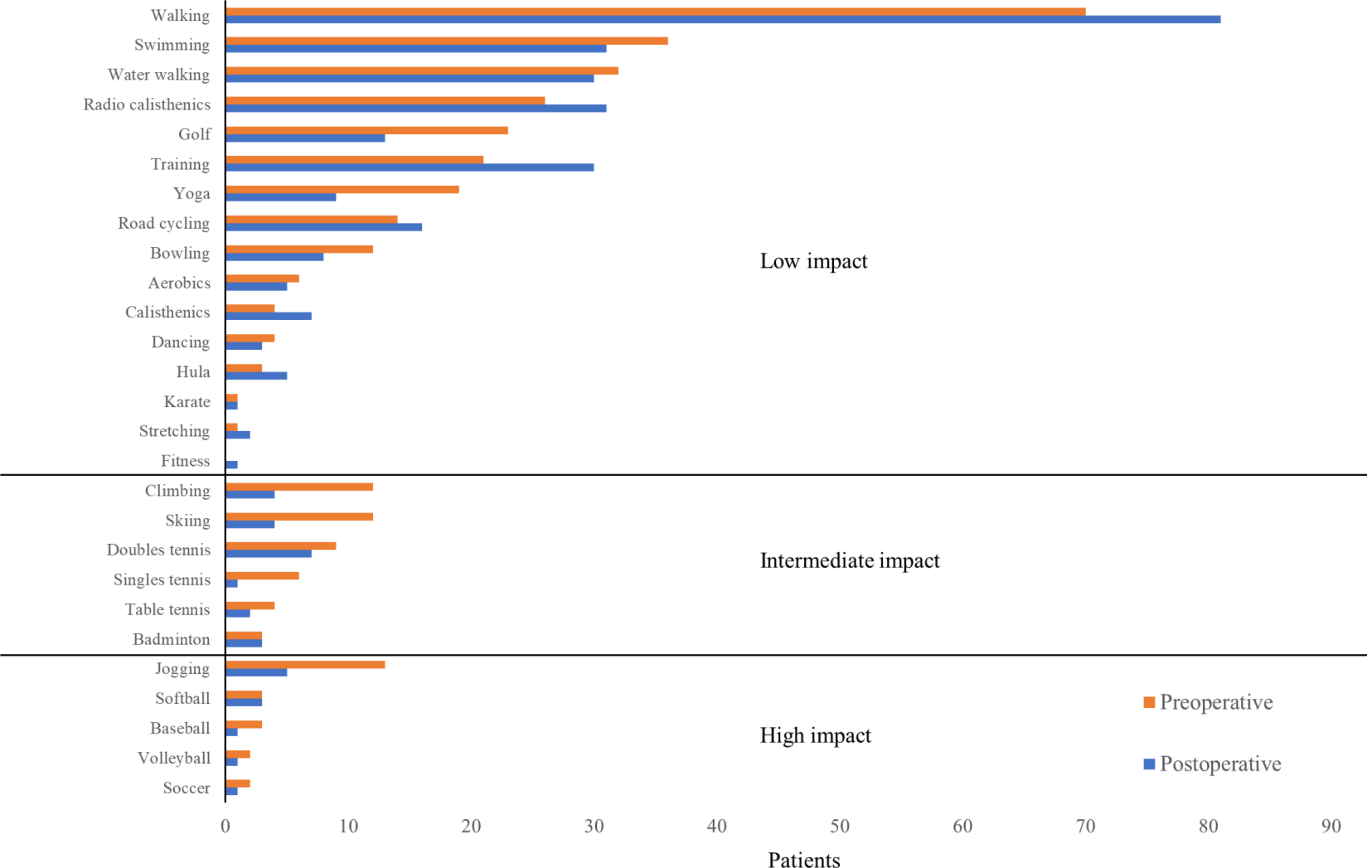
Types of sports activities participated in before and after ALS-THA (multiple answers allowed)

The types of pre- and postoperative sports activity performed are shown in Fig. 2. Regarding the impacts of the sports activity in which patients participated preoperatively, they were predominantly low-impact sports (*n* = 83, 64.9%), whereas 25 (19.5%) and 20 (15.6%) performed medium- and high-impact ones, respectively. A similar pattern was observed regarding postoperative sports activity: 110 (79.7%), 17 (12.3%) and 11 (8.0%) patients performed low-, medium- and high-impact sports activity, respectively. Patients participated in 2.7 ± 1.2 and 2.2 ± 1.2 types of sports activity pre- and postoperatively, respectively.

Patients started sports activity 7.8 ± 8.1 months from the day of surgery. Moreover, patients participated in sports activities 2.5 ± 1.9 and 2.7 ± 2.0 times per week pre- and postoperatively, respectively. The reasons for participating and not participating in sports activity are presented in Tables 1 and 2, respectively. In addition, among the 94 patients who did not participate in sports activity postoperatively, 56 (59.6%) wanted to do so, but 34 (60.7%) of them did not because of a “fear of damaging the hip joint” or “lack of confidence”.

**Table 1:** Reasons for participating in sports activity

Reasons	Patients’ ratio (%)
For health	56.9
To improve or maintain physical strength	46.0
For enjoyment or distraction	41.6
To be free of pain	38.7
Because of a lack of exercise	35.8
To move as desired	33.6
To enjoy exercise	18.2

**Table 2:** Reasons for not participating in sports activity

Reasons	Patients’ ratio (%)
Fear of damaging hip joint	42.0
Lack of confidence	35.0
Unable to move as desired	23.0
No time for sports activity	22.0
Pain	13.0
Do not enjoy sports activity	5.0

Compared with the nonparticipation group, the participation group had significantly older age, better subjective health, higher FJS and higher UCLA activity score (Table 3). Multivariate logistic regression analysis showed that age (*P* = 0.019) and UCLA activity score (*P* < 0.001) were factors significantly associated with postoperative participation in sports activity (Table 4).

**Table 3:** Univariate logistic regression analysis of factors potentially associated with postoperative participation in sports activity

Parameters	Participation group (***n*** = 137)	Nonparticipation group (***n*** = 100)	Odds ratio (95%CI)	***P-***values
Age (years)	65.3 ± 8.8	62.4 ± 10.5	1.032 (1.004–1.060)	0.025
Sex (male/female)	21/116	22/78	0.642 (0.331–1.246)	0.190
Body mass index (kg/m^2^)	23.6 ± 3.8	24.2 ± 3.4	0.957 (0.892–1.027)	0.222
Hospitalization days (day)	14.7 ± 4.7	15.7 ± 6.1	0.964 (0.919–1.012)	0.144
Preoperative range of motion (°)				
Flexion	91.0 ± 17.4	92.9 ± 18.5	0.994 (0.980–1.009)	0.423
Abduction	18.8 ± 8.6	18.5 ± 8.9	1.005 (0.976–1.035)	0.742
Adduction	10.9 ± 9.0	9.8 ± 4.9	1.025 (0.979–1.072)	0.294
External rotation	28.1 ± 12.2	29.8 ± 12.1	0.989 (0.968–1.010)	0.292
Internal rotation	15.7 ± 11.9	14.7 ± 12.7	1.007 (0.986–1.028)	0.535
Hip abductor muscle strength (%BW)				
Preoperative	20.1 ± 8.1	19.1 ± 9.7	1.014 (0.984–1.044)	0.379
Discharge	18.2 ± 6.9	17.1 ± 7.7	1.018 (0.977–1.061)	0.402
Comfortable 10-m gait speed (m/s)				
Preoperative	1.0 ± 0.3	1.0 ± 0.2	1.279 (0.460–3.561)	0.637
Discharge	0.9 ± 0.5	0.8 ± 0.2	2.585 (0.824–8.108)	0.103
Maximum 10-m gait speed (m/s)				
Preoperative	1.4 ± 0.4	1.3 ± 0.4	1.323 (0.654–2.675)	0.437
Discharge	1.2 ± 0.3	1.1 ± 0.3	2.329 (0.933–5.814)	0.070
JHEQ	60.6 ± 14.0	59.0 ± 15.3	1.008 (0.989–1.026)	0.416
Forgotten joint score (points)	71.5 ± 22.0	62.0 ± 24.9	1.017 (1.005–1.030)	0.008
EQ-5D-5L (0.01 point per increase)	0.9 ± 0.1	0.8 ± 0.2	1.024 (1.002–1.046)	0.033
EQ VAS (mm)	85.7 ± 12.1	80.1 ± 16.7	1.027 (1.006–1.049)	0.011
UCLA activity score	5.8 ± 1.8	4.1 ± 1.3	2.166 (1.627–2.883)	< 0.001

Continuous values are expressed as mean ± SD. CI, confidence interval; SD, standard deviation; JHEQ, Japanese Orthopaedic Association Hip-Disease Evaluation Questionnaire; EQ-5D-5L, EuroQol 5-dimensions 5-level; EQ VAS, EuroQol visual analogue scale; UCLA, University of California, Los Angeles

**Table 4:** Multivariate logistic regression analysis of factors potentially associated with postoperative participation in sports activity

Parameters	Odds ratio	95% confidence interval	***P***-value	
Age	1.060	1.010–1.110	0.019	
Sex	0.763	0.246–2.360	0.638	
Body mass index	1.010	0.907–1.130	0.843	
Comfortable 10-m gait speed at discharge	2.020	0.366–11.100	0.421	
Forgotten joint score	0.997	0.978–1.020	0.762	
EQ-5D-5L (0.01 point per increase)	1.000	0.970–1.030	0.925	
EQ-VAS	1.020	0.982–1.050	0.354	
UCLA activity score	1.960	1.440–2.670	< 0.001	

EQ-5D-5L, EuroQol 5-dimensions 5-level; EQ-VAS, EuroQol visual analogue scale; UCLA, University of California, Los Angeles

Regarding satisfaction with daily life and sports activity, the numbers of patients who reported being “very satisfied” or “somewhat satisfied” increased significantly after surgery (both *P* < 0.001; Table 5).

**Table 5:** Patient satisfaction with daily life and sports activity

	Daily life (%)					Sports activity (%)				
	Preoperative		Postoperative				Preoperative		Postoperative	
Very satisfied	34.4		50.0				9.0		19.8	
Somewhat satisfied	32.8		34.0				28.1		32.8	
Neither	9.8		9.8				31.1		30.5	
Somewhat dissatisfied	15.3		5.7				19.8		10.7	
Very dissatisfied	7.7		0.5				12.0		6.2	

Regarding subjective health status, there were 182 “healthy” patients (76.8%) and 55 “unhealthy” ones (23.2%). The postoperative sports activity participation rates of these two groups were 61.0% and 47.3%, respectively (*P* = 0.073). Furthermore, compared with the “unhealthy” patients, the “healthy” ones had a significantly wider preoperative hip abductor range of motion; greater pre- and postoperative hip abductor muscle strength; faster preoperative gait speed; and higher FJS, EQ-5D-5L, EQ-VAS, and UCLA activity scores upon completing the questionnaire (Table 6). In addition, logistic regression analysis showed that FJS (*P* = 0.002) and EQ-5D-5L (*P* = 0.004) were factors significantly associated with a “healthy” status (Table 7).

**Table 6:** Univariate logistic regression analysis of factors potentially associated with subjective health status

Parameters	“Healthy” (***n*** = 182)	“Unhealthy” (***n*** = 55)	Odds ratio (95%CI)	***P***-values
Age (years)	63.9 ± 9.8	64.6 ± 9.3	0.992 (0.961–1.024)	0.623
Sex (male/female)	35/147	8/47	1.399 (0.607–3.225)	0.431
Body mass index (kg/m^2^)	23.8 ± 3.6	23.9 ± 3.9	0.998 (0.991–1.083)	0.956
Hospitalization days (day)	14.9 ± 5.2	15.9 ± 5.6	0.966 (0.916–1.019)	0.200
Preoperative range of motion (°)				
Flexion	92.3 ± 17.5	90.2 ± 19.1	1.007 (0.990–1.023)	0.441
Abduction	19.3 ± 8.5	16.6 ± 9.0	1.037 (1.000–1.076)	0.048
Adduction	10.7 ± 8.2	9.5 ± 4.9	1.032 (0.972–1.096)	0.301
External rotation	29.0 ± 11.8	28.2 ± 13.4	1.006 (0.981–1.031)	0.657
Internal rotation	15.7 ± 12.0	15.9 ± 13.1	0.994 (0.970–1.019)	0.644
Hip abductor muscle strength (%BW)				
Preoperative	20.3 ± 8.6	17.6 ± 9.3	1.039 (1.000–1.079)	0.049
Discharge	17.7 ± 8.0	14.2 ± 7.5	1.069 (1.012–1.129)	0.018
Knee extension muscle strength (%BW)				
Preoperative	35.9 ± 14.2	32.9 ± 14.3	1.017 (0.994–1.040)	0.162
Discharge	29.6 ± 13.5	26.5 ± 13.0	1.014 (0.985–1.044)	0.343
Comfortable 10-m gait speed (m/s)				
Preoperative	1.0 ± 0.3	0.9 ± 0.2	4.345 (1.286–14.678)	0.018
Discharge	0.9 ± 0.5	0.8 ± 0.3	1.986 (0.538–7.333)	0.304
Maximum 10-m gait speed (m/s)				
Preoperative	1.4 ± 0.4	1.2 ± 0.4	3.441 (1.469–8.063)	0.004
Discharge	1.1 ± 0.3	1.1 ± 0.4	1.937 (0.661–5.681)	0.228
Participation rate of sports activity (%)				
Preoperative	52.7	58.2	0.802 (0.436–1.476)	0.479
Postoperative	61	47.3	1.744 (0.950–3.201)	0.073
JHEQ (points)	62.6 ± 13.6	51.2 ± 14.4	1.056 (1.032–1.081)	< 0.001
Forgotten joint score (points)	75.0 ± 18.8	47.8 ± 24.3	1.058 (1.039–1.078)	< 0.001
EQ-5D-5L(0.01 point per increase)	0.9 ± 0.1	0.7 ± 0.2	1.100 (1.065–1.137)	< 0.001
EQ VAS (mm)	87.0 ± 11.4	73.6 ± 17.4	1.067 (1.040–1.095)	< 0.001
UCLA activity score	5.4 ± 1.9	4.3 ± 1.5	1.514 (1.191–1.924)	0.001

Continuous values are expressed as mean ± SD. CI, confidence interval; SD, standard deviation; JHEQ, Japanese Orthopaedic Association Hip-Disease Evaluation Questionnaire; EQ-5D-5L, EuroQol 5-dimensions 5-level; EQ VAS, EuroQol visual analogue scale; UCLA, University of California, Los Angeles

**Table 7:** Multivariate logistic regression analysis of factors potentially associated with a “healthy” status

Parameters	Odds ratio	95% confidence interval	*P*-value
Preoperative hip abductor muscle strength	1.007	0.955–1.062	0.801
Preoperative comfortable 10-m gait speed	1.321	0.221–7.884	0.760
Forgotten joint score	1.036	1.013–1.060	0.002
EQ-5D-5L (0.01 point per increase)	1.055	1.017–1.094	0.004
UCLA activity score	1.065	0.817–1.388	0.640

EQ-5D-5L, EuroQol 5-dimensions 5-level; UCLA; University of California, Los Angeles

## Discussion

In this study, we investigated participation in sports activities and subjective health status of patients who underwent ALS-THA, and examined the factors involved with them. Overall, 54% and 57.8% of the 237 patients who underwent ALS-THA participated in sports activity pre- and postoperatively, respectively. In comparison, previous studies on THA approaches besides the ALS approach reported that 15.5–92.0% and 30.5–83.0% of patients participated in sports activity pre- and postoperatively, respectively [22–26]. Postoperative participation in sports activity is associated with characteristics such as age, the level of impact of sports activity, recommendations from physicians to participate in sports activity [14], fear of wear or dislocation of an artificial joint [27], and the physical therapy program after discharge [22, 26]. Hara et al. reported that 30.5% of Japanese patients participated in sports activity after THA via the posterior approach [23], which is lower than the rate of 57.8% after ALS-THA as reported here. In this regard, the ALS approach is reported to result in better improvement in physical function [28] and incur a lower risk of dislocation [29] than the conventional posterior approach. Therefore, the discrepancy in the rates of sport activities postoperatively may be due to the difference in the risk of early complications.

In this study, participation in low-impact sports activity, such as walking, increased postoperatively. In contrast, participation in high-impact sports activity decreased from 21 patients preoperatively to 12 postoperatively. Although there are becoming less restrictions on participation in high-impact sports activities [30], such a shift to low-impact sports activity postoperatively has been reported in several studies [22, 31]. High-impact sports activity are also reported to increase the risk of adverse events such as early loosening and excessive wear of artificial joint, although patients who participated in such activities in that study had higher physical function than those who did not [32]. Thus, the reduced postoperative participation in high-impact sports activity in the present study might reflect patients’ fears of adverse events. Further study is required to clarify the factors influencing participation in high-impact sports activity after ALS-THA.

Among patients who did not participate in sports activity postoperatively, psychological factors were more common explanations for this (60.7%) than pain or other physical factors. This is corroborated by a previous study reporting that many patients did not participate in sports activity postoperatively for psychological reasons rather than pain in the operated hip [27]. Although some patients participated in various kinds of exercise or sports activity to maintain their physical function postoperatively, psychological factors indeed hindered participation. Therefore, to increase participation in sports activity after ALS-THA, clinical staff must understand patients’ anxiety or reluctance to do so and provide evidence-based information to support them.

In this study, older age at the time of surgery and higher UCLA activity score were factors significantly associated with postoperative participation in sports activity. In contrast, several studies have reported younger age at the time of surgery as a factor linked to postoperative participation in sports activity [23, 27, 33–35]. One possible explanation for this discrepancy is that, compared with younger patients, older retired patients have more opportunities and time to participate in sports activity [36]. In our study, 22.0% of the patients who did not participate in sports activity also reported that they did not have time to do so. In some cases, older patients are more likely to participate in low-impact sports activity because there are low barriers to entry for such activities. In the current study, 85.5% of patients who participated in sports activity after surgery did so only for low-impact activities. Furthermore, a high postoperative UCLA activity score is reported to be associated with postoperative participation in sports activity [23, 27, 33–35]. Similar results were shown in Japanese patients with ALS-THA. Therefore, our results indicate that elderly Japanese patients who underwent ALS-THA with high activity levels can easily participate in sports activity postoperatively, especially low-impact ones. This finding might be useful for clinicians advising elderly patients who are concerned about participating in sports activity after ALS-THA.

Compared with patients considered “unhealthy”, FJS and EQ-5D-5L were factors significantly associated with a “healthy” status. Recently, an increasing number of studies using patient-reported outcome measures among those who have undergone THA have been reported [37, 38]. HLE increases with exercise, sports participation, maintenance of activities of daily living and high activity levels [39, 40]. Likewise, higher FJS is associated with better overall health and maintenance of activities of daily living [18, 41]. In addition, patients with higher physical function before THA are reported to have better physical function and higher activity levels after THA [24]. Thus, increasing preoperative physical function might increase the likelihood of a patient feeling “healthy” postoperatively. Specifically, as preoperative physical therapy administered by a physical therapist is effective for improving physical function [42], preoperative physical therapy is necessary to extend HLE postoperatively. In Japan, secondary OA from dysplastic hips accounts for more than 80% of cases [43], and secondary OA from hip dysplasia requires caution because the symptoms gradually worsen. Therefore, based on the results of this study, which to the best of our knowledge is the first to examine subjective health status after ALS-THA, it is possible that higher preoperative physical function is better for extended HLE after ALS-THA. As such, we suggest exercise therapy and patient education by a physical therapist before activities of daily living are restricted because of pain or deteriorated physical function due to hip OA.

The present study has some limitations. First, this study involved a self-administered questionnaire that included questions about medical history. Notably, 27.1% of patients did not reply to the questionnaire. Thus, the results might have been affected by recall and selection biases, necessitating further prospective studies to confirm the results. Second, 81.9% of the study subjects were women. Few Japanese patients are obese, and BMI values are often close to ideal values. In addition, Japanese patients undergoing THA are often due to secondary hip osteoarthritis. Although this sex ratio reflects the higher incidence and progression of OA in Japanese women than in men [44], the results might not be generalizable to other populations. Third, we did not survey some socio-economic factors including working status, educational history and income circumstances etc. Our results suggest that older retired patients may have relatively more opportunities and free time to participate in sports activities, and their impact on participation in sports activities should be further investigated. Finally, this study was conducted prior to the COVID-19 pandemic; Clement et al. reported that the pandemic increased the waiting period for surgery, increased the preoperative “worse than death” status and decreased the quality of life [45]. Therefore, further research on sports activity and health status, including quality of life, during the pandemic is warranted.

## Conclusion

Among Japanese patients who underwent ALS-THA, 54.0% and 57.8% participated in sports activity pre- and postoperatively, respectively. Older age and UCLA activity score were factors significantly associated with postoperative participation in sports activity. Furthermore, 76.8% of patients considered “healthy”, and FJS and EQ-5D-5L were factors significantly associated with a “healthy” status. These findings could be useful for advising elderly patients concerned about participating in sports activity and their health after ALS-THA.

## Electronic supplementary material

Below is the link to the electronic supplementary material.

Supplementary Material 1

## Data Availability

The datasets used and/or analysed during the current study are available from the corresponding author on reasonable request.

## List of abbreviations

ALS-THA: Total hip arthroplasty via the anterolateral approach in the supine position
EQ-5D-5L: EuroQol 5-dimensions 5-level
EQ-VAS: EuroQol Visual Analogue Scale
FJS: Forgotten Joint Score
HLE: Healthy life expectancy
JHEQ: Japanese Orthopaedic Association Hip-Disease Evaluation Questionnaire
OA: Hip osteoarthritis
SD: Standard deviation
THA: Total hip arthroplasty
UCLA: University of California Los Angeles
