# Supplementary material for: Sports activity participation and subjective health status of patients after total hip arthroplasty via the anterolateral-supine approach: a case series study
Source: BMC Musculoskelet Disord. 2022 Oct 29;23:943. doi: 10.1186/s12891-022-05886-6 (PMC9617043; doi:10.1186/s12891-022-05886-6)
Supplement: Supplementary file 1 — Supplementary Material 1 [file 12891_2022_5886_MOESM1_ESM.docx]

**Questionnaire on sports activities after total hip arthroplasty**

Please read the enclosed instructions before you fill out this questionnaire. Please provide responses in the blank spaces provided, circle the items in parentheses and check the boxes that apply to you. This questionnaire is 7 pages long and should take about 15 minutes to complete. The term ‘sports activities’ refers to any exercise performed voluntarily. If you have had multiple surgeries, please provide answers with reference to the last surgery you had. If you agree to the secondary use of your data, please check the box below to provide consent.

We apologize for taking up your time but sincerely appreciate your cooperation.

□ I agree to the secondary use of my data.

Date / /

**[Basic information]**

1. Name:

2. Age:

3. Sex: ( Male / Female )

4. Date of operation: / /

5. Do you currently have any problems that affect your daily life? ( Yes / No )

6. If you answered “yes” to Question 5, please answer the following: How do these problems affect you? Please check *all* the boxes that apply.

| □　Activities of daily living | □　Going out | □　Exercise (including sports) |
| --- | --- | --- |
| □　Job, housework or schoolwork (limited time, work, etc.) | | □　Other (　　　　　　　) |

7. What is your current health condition? Please check *one* of the boxes that applies to you.

| □　Good | □　Fairly good | □　Normal | □　Not Good | □　Bad |
| --- | --- | --- | --- | --- |

8. Are you certified as needing nursing care?

( No / Requiring assistance / Requiring nursing care / I don't want to answer )

**[Information before surgery]**

9. Did you participate in sports activities before surgery? ( Yes / No )

10. Please indicate your satisfaction with your daily activities and sports activities.

|  | Extremely dissatisfied | Somewhat dissatisfied | Neither | Somewhat satisfied | Very satisfied |
| --- | --- | --- | --- | --- | --- |
| Satisfaction with daily activities | □ | □ | □ | □ | □ |
| Satisfaction with sports activities | □ | □ | □ | □ | □ |

**If you answered “yes” to Question 9, please answer Questions 11 and 12; otherwise, please skip to Question 13.**

11. Please check the boxes of the sports activities that you participated in (check *all* that apply).

| □ Singles tennis | □ Baseball | □ Road cycling | □ Soccer |
| --- | --- | --- | --- |
| □ Doubles tennis | □ Yoga | □ Softball | □ Table tennis |
| □ Walking | □ Golf | □ Badminton | □ Skiing |
| □ Water walking | □ Bowling | □ Volleyball | □ Swimming |
| □ Radio calisthenics | □ Jogging | □ Training | □ Climbing |

Other:

12. How often did you participate in sports activities? Please answer for each category.

Type: Frequency: times/month, times/week

Type: Frequency: times/month, times/week

Type: Frequency: times/month, times/week

**[Information after surgery]**

13. Did you participate in sports activities after surgery? ( Yes / No )

**If you answered “yes” to Question 13, please answer Questions 14–17; otherwise please skip to Question 18.**

14. Please check the boxes of the sports activities that you participated in (check *all* that apply).

| □ Singles tennis | □ Baseball | □ Road cycling | □ Soccer |
| --- | --- | --- | --- |
| □ Doubles tennis | □ Yoga | □ Softball | □ Table tennis |
| □ Walking | □ Golf | □ Badminton | □ Skiing |
| □ Water walking | □ Bowling | □ Volleyball | □ Swimming |
| □ Radio calisthenics | □ Jogging | □ Training | □ Climbing |

Other:

15. How often did you participate in sports activities? Please answer for each category.

Type: Frequency: times/month, times/week

Type: Frequency: times/month, times/week

Type: Frequency: times/month, times/week

16. When did you start participating in sports activities after surgery?

years and months after surgery

17. Why did you choose to participate in sports activities? (check *all* that apply)

| □ Lack of exercise | □ Liking exercise | □ Enjoyment or distraction | □ Health |
| --- | --- | --- | --- |
| □ No movement impediments | □ No pain | □ Physical strength or maintenance | |

Other:

**If you answered “no” to Question 13, please answer Questions 18–20; otherwise please skip to Question 21.**

18. Why did you *not* participate in sports activities? (check *all* that apply)

| □ Unable to move as desired | □ Pain | □ Advice from doctor | □ Health |
| --- | --- | --- | --- |
| □ Fear of damage to the hip | □ No confidence | □ Not interested in exercise | |

Other:

19. Do you hope to participate in sports activities in the future? ( Yes / No )

20. If you answered “yes” to Question 19, please choose the type(s) of sport activities that you would like to participate in (check *all* that apply).

| □ Singles tennis | □ Baseball | □ Road cycling | □ Soccer |
| --- | --- | --- | --- |
| □ Doubles tennis | □ Yoga | □ Softball | □ Table tennis |
| □ Walking | □ Golf | □ Badminton | □ Skiing |
| □ Water walking | □ Bowling | □ Volleyball | □ Swimming |
| □ Radio calisthenics | □ Jogging | □ Training | □ Climbing |

Other:

**[Current information]**

21. Please indicate your current satisfaction with your daily activities and sports activities.

|  | Extremely dissatisfied | Somewhat dissatisfied | Neither | Somewhat satisfied | Very satisfied |
| --- | --- | --- | --- | --- | --- |
| Satisfaction with daily activities | □ | □ | □ | □ | □ |
| Satisfaction with sports activities | □ | □ | □ | □ | □ |

22. We would like to ask you about your current level of activity. Please circle the number of the item that best reflects your status in the following table.

| Level | Activity |
| --- | --- |
| 10. | Regularly participate in impact sports such as jogging, tennis skiing, acrobatics, ballet, heavy labor, or backpacking |
| 9. | Sometimes participate in impact sports |
| 8. | Regularly participate in very active events, such as bowling or golf |
| 7. | Regularly participate in active events, such as bicycling |
| 6. | Regularly participate in moderate activities, such as swimming and unlimited housework or shopping |
| 5. | Sometimes participate in moderate activities |
| 4. | Regularly participate in mild activities, such as walking, limited housework, and limited shopping |
| 3. | Sometimes participate in mild activities |
| 2. | Mostly inactive: restricted to minimal activities of daily living |
| 1. | Wholly inactive: dependent on others; cannot leave residence |

23. How aware are you of your hip joint in everyday life? Please select one answer for each item by ticking the appropriate box.

|  |  | Never | Almost never | Seldom | Sometimes | Mostly |
| --- | --- | --- | --- | --- | --- | --- |
| 1. | In bed at night | □ | □ | □ | □ | □ |
| 2. | When you are sitting on a chair for more than 1 hour | □ | □ | □ | □ | □ |
| 3. | When you are walking for more than 15 minutes | □ | □ | □ | □ | □ |
| 4. | When you are taking a bath/shower | □ | □ | □ | □ | □ |
| 5. | When you are traveling in a car | □ | □ | □ | □ | □ |
| 6. | When you are climbing stairs | □ | □ | □ | □ | □ |
| 7. | When you are walking on uneven ground | □ | □ | □ | □ | □ |
| 8. | When you are standing up from a low-sitting position | □ | □ | □ | □ | □ |
| 9. | When you are standing for long periods of time | □ | □ | □ | □ | □ |
| 10. | When you are doing housework or gardening | □ | □ | □ | □ | □ |
| 11. | When you are taking a walk/hiking | □ | □ | □ | □ | □ |
| 12. | When you are doing your favorite sport | □ | □ | □ | □ | □ |

24. Do you experience hip pain or discomfort in your daily life or during sports (for those who do them)? Please circle the one that applies most to you.

Daily life: ( None / Pain / Discomfort / Both )

Sports activities: ( None / Pain / Discomfort / Both )

25. We would like to ask you about your current health status. For each of the following questions, please check the box that best applies.

| MOBILITY |  |
| --- | --- |
| I have no problems in walking about | ❑ |
| I have slight problems in walking about | ❑ |
| I have moderate problems in walking about | ❑ |
| I have severe problems in walking about | ❑ |
| I am unable to walk about | ❑ |
| SELF-CARE |  |
| I have no problems washing or dressing myself | ❑ |
| I have slight problems washing or dressing myself | ❑ |
| I have moderate problems washing or dressing myself | ❑ |
| I have severe problems washing or dressing myself | ❑ |
| I am unable to wash or dress myself | ❑ |
| USUAL ACTIVITIES (e. g. work, study, housework, family or leisure activities) | |
| I have no problems doing my usual activities | ❑ |
| I have slight problems doing my usual activities | ❑ |
| I have moderate problems doing my usual activities | ❑ |
| I have severe problems doing my usual activities | ❑ |
| I am unable to do my usual activities | ❑ |
| PAIN / DISCOMFORT |  |
| I have no pain or discomfort | ❑ |
| I have slight pain or discomfort | ❑ |
| I have moderate pain or discomfort | ❑ |
| I have severe pain or discomfort | ❑ |
| I have extreme pain or discomfort | ❑ |
| ANXIETY / DEPRESSION |  |
| I am not anxious or depressed | ❑ |
| I am slightly anxious or depressed | ❑ |
| I am moderately anxious or depressed | ❑ |
| I am severely anxious or depressed | ❑ |
| I am extremely anxious or depressed | ❑ |

The best health you can imagine

26. We would like to know how good or bad your health is TODAY.

This scale is numbered from 0 to 100.

100 means the best health you can imagine.

0 means the worst health you can imagine.

Mark an X on the scale to indicate how your health is TODAY.

Now, please write the number you marked on the scale in the box below.

YOUR HEALTH TODAY =

The best health you can imagine

This concludes the survey.

**Thank you very much for your responses!**

Please send your completed questionnaire in the return envelope provided.

If you have any comments or questions about this survey, please write them in the box below.

Thank you for your cooperation.
